# miR-410 and miR-495 Are Dynamically Regulated in Diverse Cardiomyopathies and Their Inhibition Attenuates Pathological Hypertrophy

**DOI:** 10.1371/journal.pone.0151515

**Published:** 2016-03-21

**Authors:** Amanda L. Clark, Sonomi Maruyama, Soichi Sano, Anthony Accorsi, Mahasweta Girgenrath, Kenneth Walsh, Francisco J. Naya

**Affiliations:** 1 Department of Biology, Program in Cell and Molecular Biology, Boston University, Boston, Massachusetts, United States of America; 2 Whitaker Cardiovascular Institute, Boston University School of Medicine, Boston, Massachusetts, United States of America; 3 Health Sciences Department, College of Health and Rehabilitation Sciences, Boston University, Boston, Massachusetts, United States of America; University of Western Ontario, CANADA

## Abstract

Noncoding RNAs have emerged as important modulators in cardiac development and pathological remodeling. Recently, we demonstrated that regulation of the *Gtl2-Dio3* noncoding RNA locus is dependent on the MEF2 transcription factor in cardiac muscle, and that two of its encoded miRNAs, miR-410 and miR-495, induce robust cardiomyocyte proliferation. Given the possibility of manipulating the expression of these miRNAs to repair the damaged heart by stimulating cardiomyocyte proliferation, it is important to determine whether the *Gtl2-Dio3* noncoding RNAs are regulated in cardiac disease and whether they function downstream of pathological cardiac stress signaling. Therefore, we examined expression of the above miRNAs processed from the *Gtl2-Dio3* locus in various cardiomyopathies. These noncoding RNAs were upregulated in all cardiac disease models examined including myocardial infarction (MI) and chronic angiotensin II (Ang II) stimulation, and in the cardiomyopathies associated with muscular dystrophies. Consistent with these observations, we show that the *Gtl2-Dio3* proximal promoter is activated by stress stimuli in cardiomyocytes and requires MEF2 for its induction. Furthermore, inhibiting miR-410 or miR-495 in stressed cardiomyocytes attenuated the hypertrophic response. Thus, the *Gtl2-Dio3* noncoding RNA locus is a novel marker of cardiac disease and modulating the activity of its encoded miRNAs may mitigate pathological cardiac remodeling in these diseases.

## Introduction

Cardiovascular disease is the leading cause of death and morbidity in the developed world. Elucidating the underlying gene regulatory mechanisms that lead to heart failure could uncover new ways to develop novel therapeutic strategies for cardiovascular disease. Recently, microRNAs (miRNAs) have been shown to play roles in cardiac hypertrophy and heart failure. Accumulating evidence suggests that manipulating miRNA expression is a potential therapeutic approach in the treatment of cardiovascular disease [1–4].

The *Gtl2-Dio3* locus harbors one of the largest known noncoding RNA clusters in mammals. This locus generates more than 50 miRNAs, small nucleolar RNAs, and one or more long noncoding RNAs (lncRNAs) including *Gtl2 (MEG3* in humans), which resides at the 5’-end of the putative, single (~200 kilobase) polycistronic transcript [5,6]. Expression of the *Gtl2-Dio3* locus has been shown to correlate with induced and embryonic stem cell pluripotency, and its dysregulation is associated with a number of human diseases [7–11].

We previously demonstrated that the *Gtl2-Dio3* locus is coordinately regulated by the MEF2 transcription factor in skeletal muscle differentiation and regeneration [12]. More recently, we have shown that the *Gtl2-Dio3* locus is also regulated by MEF2 in cardiac muscle, and that a subset of its encoded miRNAs, miR-410 and miR-495, is able to induce proliferation in neonatal cardiomyocytes *in vitro* [13]. Because of their ability to promote proliferation of differentiated cardiomyocytes and the potential of harnessing this activity to promote cardiac regeneration, we were interested in examining their regulation the diseased heart. Additionally, given that MEF2 is a key mediator of pathological remodeling of the heart [14], we were interested in determining whether silencing of its downstream miRNA targets is capable of modulating the response to stress signaling in cardiomyocytes.

Here, we performed a comprehensive expression analysis of a subset of *Gtl2-Dio3* miRNAs in mouse models of myocardial infarction (MI) and pathological hypertrophy induced by the hypertensive agonist angiotensin II (Ang II). Moreover, we examined cardiac expression of *Gtl2-Dio3* miRNAs in the *mdx* mouse model of Duchenne Muscular Dystrophy (DMD) and the *DyW* mouse model of laminin-α2 (merosin) deficient congenital muscular dystrophy type 1A (MDC1A), degenerative skeletal muscle diseases that have associated cardiomyopathy. Although the aforementioned cardiac disease models have distinct etiologies the *Gtl2-Dio3* miRNAs were upregulated in all of these cardiomyopathies. Finally, we show for the first time that knockdown of selected *Gtl2-Dio3* miRNAs in cardiomyocytes subjected to stress stimuli *in vitro* attenuates the maladaptive increase in cell size, indicating that these noncoding RNAs are essential mediators of pathological signaling in the heart.

## Materials and Methods

### Mouse Models

Cardiac tissue for myocardial infarction (MI) and angiotensin II-treated (Ang II) cardiomyopathy models were performed as described previously [15,16]. For the MI model, permanent left anterior descending coronary artery ligation was performed. For the Ang II model, human angiotensin II (Sigma-Aldrich) was administered to mice subcutaneously by osmotic pump (2mg/kg/day). MEF2A knockout mice were generated as previously described [17]. C57BL/10ScSn-mdx/J (*mdx*) mice were purchased from The Jackson Laboratory. Laminin-α2-knockout (*DyW*) tissue have been described previously [18].

### Isolation of NRVMs

Ventricles from neonatal rats were isolated from approximate 10 1-day-old Sasco Sprague-Dawley neonatal rats (Charles River Laboratories). Briefly, whole hearts were harvested, and ventricles were isolated from the atria and transferred to pre-chilled 1X Hanks’ balanced salt solution/0.025% trypsin and incubated overnight at 4°C. The following day, digestion was performed by adding 10mg/mL collagenase II (Worthington) to isolate individual cardiomyocytes. Cells were pre-plated on uncoated 10-cm dishes to remove fibroblasts. Cells were plated in antibiotic-free growth medium at a density of 4 x 10^6^ cells/10-cm dish on gelatinized dishes. After 24 hours in culture, cells were washed with 1X PBS and switched to 0.5X Nutridoma-SP (Roche) in DMEM, a low-serum medium.

### Plasmids and miRNA Inhibitors

The mouse *Gtl2* promoter (0.5kb) containing the MEF2 binding site was cloned into pGL3-Basic (Promega) as previously described [12]. miRNA inhibitors (antimiRs) hsa-miR-410-3p, hsa-miR-495-3p, and hsa-433-3p were purchased from Dharmacon.

### Quantitative RT-PCR

RNA from cardiac muscle or NRVM experiments (n≥3) was used to synthesize cDNA using reverse transcriptase with random hexamers according to the instructions of the manufacturer (Promega). cDNAs for miRNA expression were synthesized using the TaqMan miRNA reverse transcriptase kit (Applied Biosystems) for detection of mature miRNAs as previously described [13]. Quantitative RT-PCR was performed in triplicate using Power SYBR Green Master Mix (Applied Biosystems) with a 7900HT sequence detection system (Applied Biosystems). The primers used were 5S rRNA stem loop forward 5’-GTTGGCTCTGGTGCAGGGTCCGAGGTATTCGCACCAGAGCCAACAAAGCC, miR-410 stem loop 5’-GTTGGCTCTGGTGCAGGGTCCGAGGTATTCGCACCAGAGCCAACACAGGC, miR-495 stem loop 5’-GTTGGCTCTGGTGCAGGGTCCGAGGTATTCGCACCAGAGCCAACAAGAAG, 5S rRNA forward 5’-GAATACCGGGTGCTGTAGGC, miR-410 forward 5’-CCGCCAATATAACACAGATGGCC, miR-495 forward 5’-GCCAAACAAACATGGTGCACTT, *Gapdh* forward 5’-TGGCAAAGTGGAGATTGTTGCC and reverse 5’-AAGATGGTGATGGGCTTCCCG, *Nppa* forward 5’- ACCTGCTAGACCACCTGGAGGAG and reverse 5’- CCTTGGCTGTTATCTTC-GGTACCGG, *Nppb* forward 5’- ATCTCCAGAAGGTGCTGCCCCAG and reverse 5’- CGCGGTCTTCCTAAAACAACCTCAG, *Gtl2* forward 5’-TTTGATCACTGTCTCCAGCCTGCTG and reverse 5’-GATGATGAGACTTCCGACCAGCCA.

### MiRNA Transfection

miRNA inhibitors (Dharmacon) were transfected into NRVMs using a standard reverse transfection protocol at a final concentration of 50nM. Briefly, Lipofectamine RNAiMAX transfection reagent (Life Technologies) was diluted in Opti-MEM (Life Technologies) and added to the miRNA inhibitors. Cells were seeded 30 minutes later.

### Luciferase Assays

Cells were harvested for luciferase activity 48 hours after transfection. Cells were lysed in 1X passive lysis buffer (Promega). To measure luciferase, LARII (Promega) was normalized by *Renilla* luciferase assay (Promega). All luciferase assays were performed in triplicate (n≥3).

### Cell Culture Immunofluorescence

Cells were cultured on sterilized coverslips coated with Matrigel. Cells were fixed in 4% paraformaldehyde and blocked in 3% BSA (Promega) for 1 hour at room temperature. Cells were incubated in primary antibody [anti-α-actinin (1:500, Sigma)] diluted in antibody dilution buffer (1X PBS/1% BSA/0.3% Triton X-100) overnight at 4°C. The following day, cells were washed in 1X PBS and incubated with fluorochrome-conjugated antibodies diluted in antibody dilution buffer. Secondary antibody included Alexa Fluor 488 donkey anti-mouse heavy + light chain (1:200, Invitrogen). Cells were washed in 1X PBS and mounted on slides with Vectashield mounting medium with DAPI (Vector Laboratories). Slides were sealed with nail polish and stored at 4°C protected from light. Immunofluorescence images were taken with an Olympus Disk Scanning Unit spinning disk confocal microscope.

### Statistical Analysis

All numerical quantifications are representative of the mean ± S.E. of at least three experiments performed independently. Statistically significant differences between two populations of data were determined using Student’s *t* test. Appropriate data sets were also analyzed for significance using 2-way ANOVA. *p* ≤ 0.05 was considered to be statistically significant.

### Ethics statement

Experimental procedures on animals used in this study were reviewed and approved by the Institutional Animal Care and Use Committees (IACUC) of Boston University (protocol number 13–048). These studies were conducted in accordance with the principles of animal care and experimentation in the Guide For the Care and Use of Laboratory Animals.

## Results

### *Gtl2-Dio3* miRNAs are dynamically regulated in cardiac injury and hypertrophy mouse models

Recently, we reported that two miRNAs processed from the *Gtl2-Dio3* noncoding RNA locus, miR-410 and miR-495, potently stimulated proliferation of neonatal cardiomyocytes *in vitro* [13]. Because mature cardiomyocytes are largely unable to proliferate in the heart in response to damage or disease, we were interested in determining whether expression of the *Gtl2-Dio3* miRNAs is regulated in adult cardiomyopathies.

**Fig 1:**
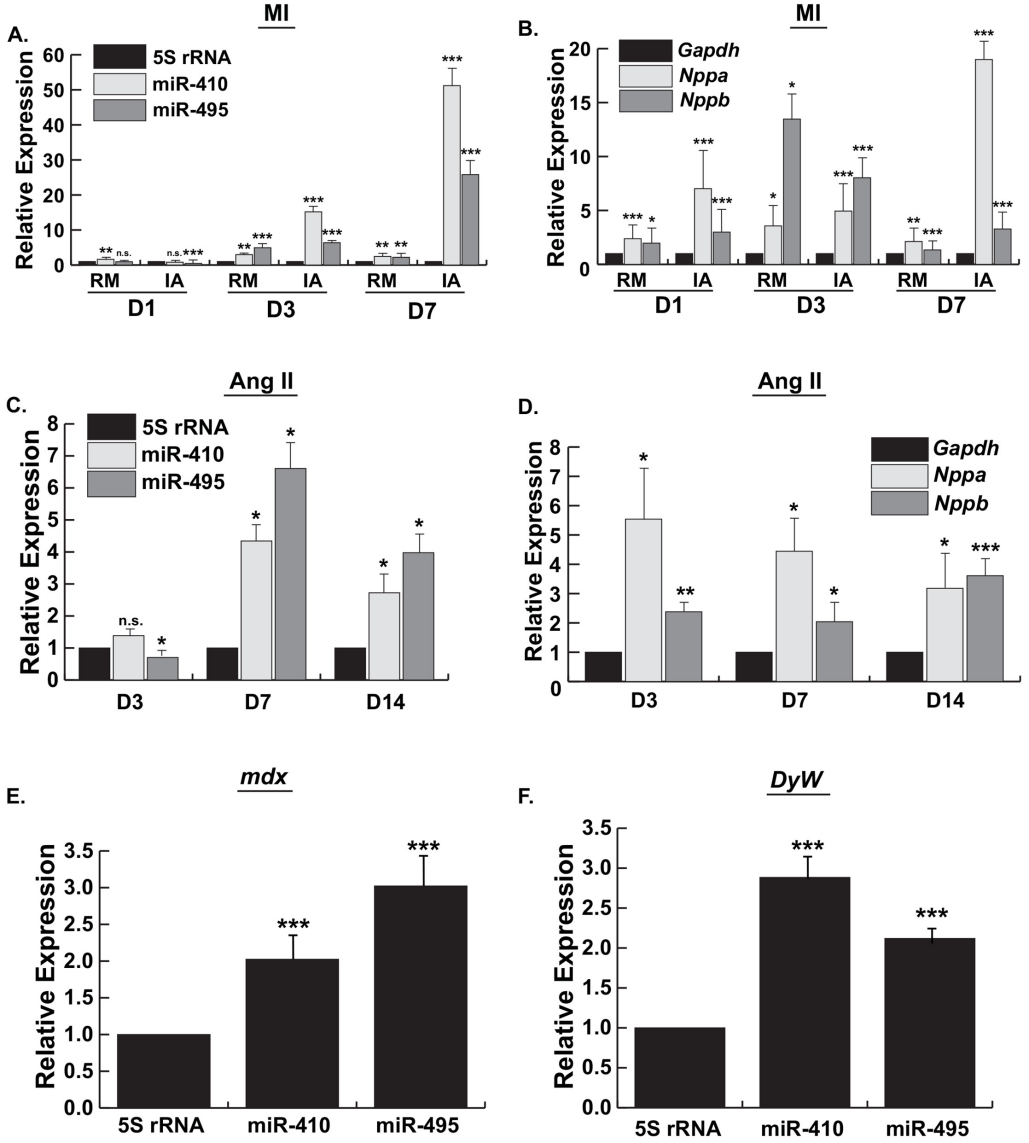
*Gtl2-Dio3* miRNAs are dynamically regulated in diverse cardiomyopathies. *A and B*, Quantitative RT-PCR expression of miR-410 and miR-495 (*A*), and the hypertrophy marker genes *Nppa* and *Nppb* (*B*), 1, 3, and 7 days post-myocardial infarction (MI) compared to sham-operated control. Values normalized to 5S RNA. RM, remote area; IA, infarcted area. *C and D*, Quantitative RT-PCR expression of miR-410 and miR-495 (*C*), and *Nppa* and *Nppb* (*D*), in hearts treated with angiotensin II (Ang II) for 3-, 7-, and 14-days. *E and F*, Quantitative RT-PCR expression of miR-410 and miR-495 in 10 week old *mdx* hearts (*E*) and *DyW laminin*-α2 knockout hearts (*F*). Error bars represent mean ± S.E. *n*.*s*., not significant; *, *p*<0.05; **, *p*<0.01; ***, *p*<0.001.

Initially, we examined expression of *Gtl2-Dio3* noncoding RNAs in a heart injury model by surgically inducing myocardial infarction (MI) in mice. We examined temporal expression of the *Gtl2-Dio3*-encoded miRNAs, miR-410 and miR-495, in the progression of this cardiac injury at 1-, 3-, and 7-days post infarction. Moreover, we compared their spatial expression differences in remote (RM), i.e. uninjured or spared myocardium, and infarcted (IA) regions of the MI heart. As shown in Fig 1A, one day after infarction there was a modest though variable dysregulation of the miRNAs in both the RM and IA. By 3 days post-injury, however, expression of both miR-410 and miR-495 was significantly upregulated in the remote and infarcted areas of the heart, but to a significantly greater extent in the IA relative to RM. By 7 days post-infarct, expression of these *Gtl2-Dio3* miRNAs in the RM remained significantly upregulated compared to sham but at levels comparable to those observed on day 3. Notably, expression of miR-410 and miR-495 in the IA on day 7 was now 10-20-fold higher relative to the RM levels (Fig 1A). To monitor potential spatio-temporal differences in a pathological remodeling process in these samples we examined the expression of the hypertrophic marker genes *Nppa* and *Nppb*. Both *Nppa* and *Nppb* were induced as early as day 1 in both the RM and IA, and with the exception of *Nppa* expression in the IA on day 7, did not display substantial differences in expression between these two regions (Fig 1B). Together, these results reveal a temporal and region-specific regulation of the *Gtl2-Dio3* miRNAs in the progression of ischemic heart injury.

To determine whether the *Gtl2-Dio3* noncoding RNAs are regulated in response to pathological cardiac hypertrophy induced by a chronic neurohormonal insult, we also examined the expression of these transcripts 3-, 7-, and 14-days after administration of angiotensin II (Ang II), a potent cardiotoxic hormone that promotes extensive myocardial hypertrophy and fibrosis [19]. Similar to the temporal expression differences observed in MI, there was variable dysregulation of miR-410 and -495 in the early stages of Ang II-mediated hypertrophy on day 3 (Fig 1C). However, by 7 days of chronic Ang II treatment the miRNAs were significantly upregulated relative to sham controls. The *Gtl2-Dio3* miRNAs remained significantly upregulated at 14 days post Ang II treatment (Fig 1C). Like the infarcted heart, expression of *Nppa* and *Nppb* were significantly induced early in the pathology and remained upregulated throughout the course of the insult (Fig 1D). These data indicate that the *Gtl2-Dio3* noncoding RNAs are also dynamically regulated in response to a cardiotoxic insult.

### *Gtl2-Dio3* miRNAs are upregulated in dystrophic cardiomyopathies

To determine whether the *Gtl2-Dio3* noncoding RNAs are regulated in cardiomyopathies stemming from mutations in genes encoding proteins necessary for the structural integrity of striated muscle, we examined their expression in hearts from two mouse models of muscular dystrophy. Dystrophin-deficient *mdx* mice are the mouse model of Duchenne Muscular Dystrophy (DMD). As shown in Fig 1E, expression of the *Gtl2-Dio3* noncoding RNAs in adult hearts (10 week old) from *mdx* mice, the mouse model of Duchenne Muscular Dystrophy (DMD), was significantly upregulated. We next examined *Gtl2-Dio3* expression in laminin-α2 (merosin)-deficient (*DyW*) mice, a model of Congenital Muscular Dystrophy (MDC1A). Like *mdx* hearts, expression of the *Gtl2-Dio3* noncoding RNAs in 7 week old laminin-α2-deficient hearts was significantly upregulated (Fig 1F). Together, these data indicate the *Gtl2-Dio3* miRNAs are upregulated in dystrophic cardiomyopathies arising from structural abnormalities.

### *Gtl2-Dio3* miRNAs are induced in hypertrophic cardiomyocytes and their inhibition attenuates hypertrophy *in vitro*

Based on the upregulation of *Gtl2-Dio3* noncoding RNAs in various cardiac disease models, we next wanted to determine whether their upregulation in diseased hearts reflects their activation in cardiomyocytes and whether they are required for promoting the stress response in cardiomyocytes.

**Fig 2:**
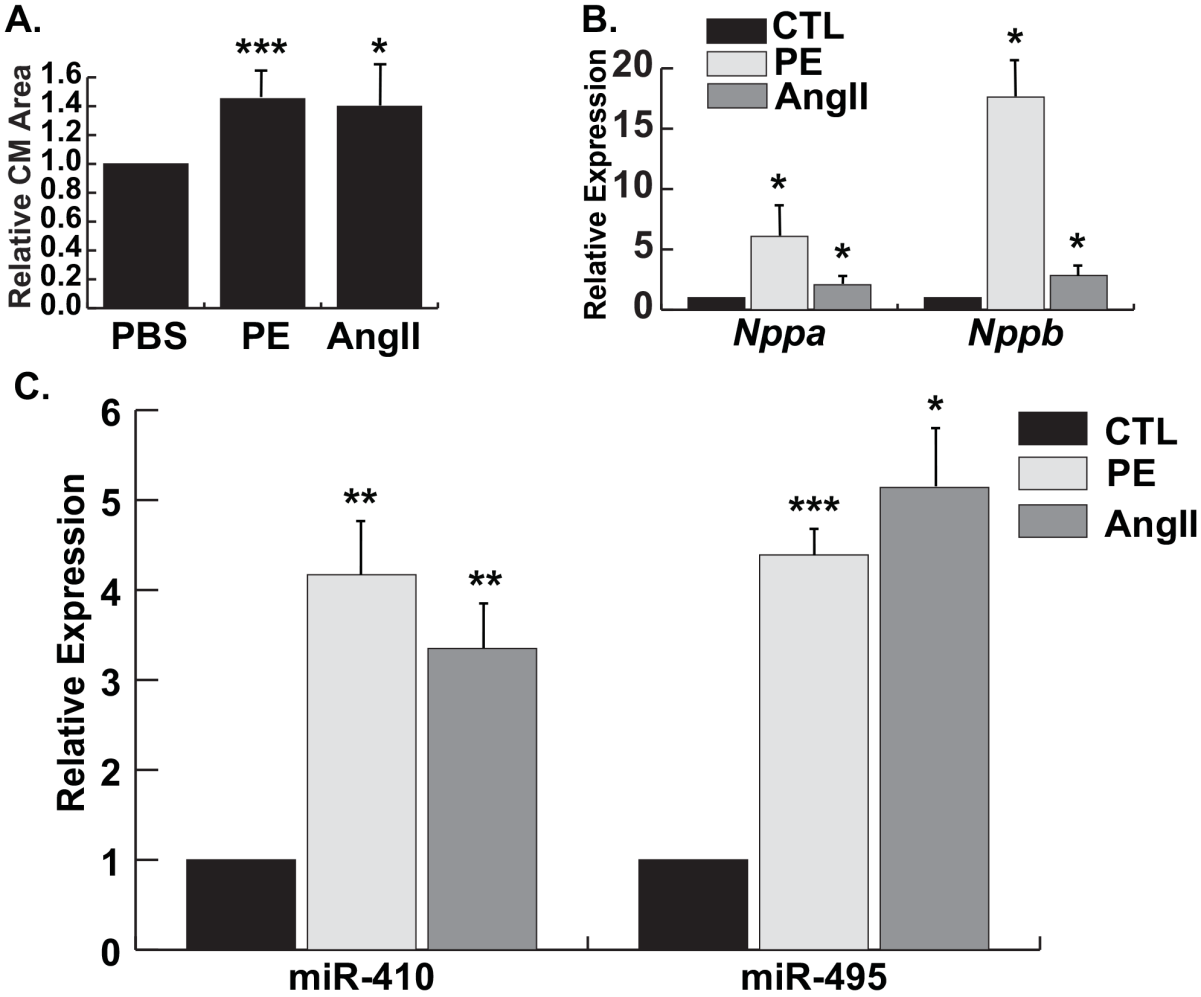
*Gtl2-Dio3* miRNAs are upregulated *in vitro* in cardiac hypertrophy. *A*, Relative cardiomyocyte (CM) area in control (PBS), phenylephrine-treated (PE), and angiotensin II-treated (Ang II) NRVMs. *B*, Quantitative RT-PCR expression of the hypertrophic markers *Nppa* and *Nppb* in PE- and Ang II-treated NRVMs. Values are normalized to PBS control (CTL). *C*, Quantitative RT-PCR expression of miR-410 and miR-495 in PE- and AngII-treated NRVMs, respectively. Error bars represent mean ± S.E. *, *p*<0.05; **, *p*<0.01; ***, *p*<0.001.

Initially, we examined expression of *Gtl2-Dio3* noncoding RNAs in stressed cardiomyocytes by subjecting NRVMs to hypertrophic stimuli. NRVMs were treated with either Ang II or phenylephrine (PE), another hormonal cardiac stressor that promotes pathological cardiomyocyte hypertrophy. As expected, both Ang II and PE induced cardiac hypertrophy (Fig 2A and 2B). Similar to our *in vivo* data, we found that the *Gtl2-Dio3* miRNAs were significantly upregulated in both hypertrophic conditions (Fig 2C). Thus, their upregulated expression in the various cardiomyopathies examined likely stems from their induction in cardiomyocytes.

**Fig 3:**
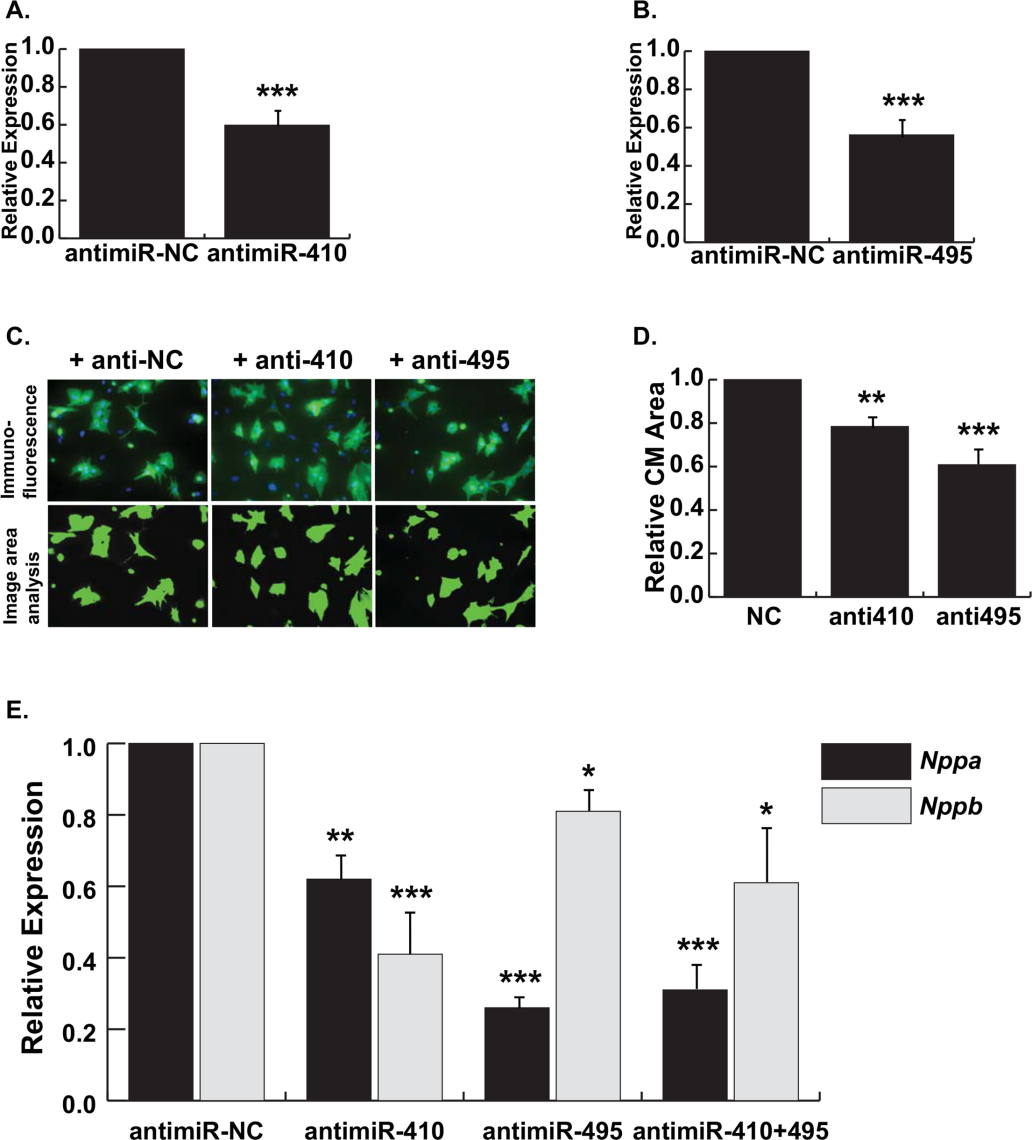
Knockdown of miR-410 and miR-495 results in decreased cardiomyocyte hypertrophy. *A*, Quantitative RT-PCR expression of miR-410 in NRVMs transfected with antimiR-410. *B*, Quantitative RT-PCR expression of miR-495 in NRVMs transfected with antimiR-495. *C*, Representative images of PE-treated NRVMs transfected with antimiR-NC, antimiR-410, and antimiR-495. *Top panels*, Immunofluorescence images of NRVMs stained with α-actinin (green) and Dapi (blue). *Bottom panels*, Computer generated image area analysis of cardiomyocytes. *D*, Relative CM area in NRVMs transfected with antimiR-410 and antimiR-495 compared to antimiR-NC shows significantly reduced cell size. *E*, Quantitative RT-PCR expression of *Nppa* and *Nppb* in PE-treated NRVMs transfected with antimiR-410, anti-miR-495, or both (anti-miR-410 + miR-495) compared to antimiR-NC. Error bars represent mean ± S.E. *, *p*<0.05; **, *p*<0.01; ***, *p*<0.001.

The upregulated expression of *Gtl2-Dio3* noncoding RNAs in the various cardiac disease models strongly suggests a role in pathological cardiac remodeling pathways. In order to determine the requirement of *Gtl2-Dio3* miRNAs in pathological signaling, we inhibited their expression in NRVMs treated with PE since this hormone induced the most robust hypertrophic effect. We first confirmed that addition of miRNA-specific antimiRs significantly decreased expression of miR-410 and miR-495, respectively (Fig 3A and 3B). Knockdown of these miRNAs individually in PE-treated NRVMs resulted in significantly decreased cardiomyocyte area and expression of the hypertrophic marker genes *Nppa* and *Nppb* (Fig 3C and 3D). Combinatorial knockdown of miR-410 and miR-495 did not further blunt hypertrophy as determined by *Nppa* and *Nppb* expression (Fig 3E). Together, these results indicate that inhibition of either miR-410 or miR-495 is sufficient to attenuate the hypertrophic response of cardiomyocytes exposed to stress stimuli suggesting they function as prohypertrophic miRNAs.

### *Gtl2-Dio3* miRNAs are regulated by MEF2 in cardiac stress signaling

**Fig 4:**
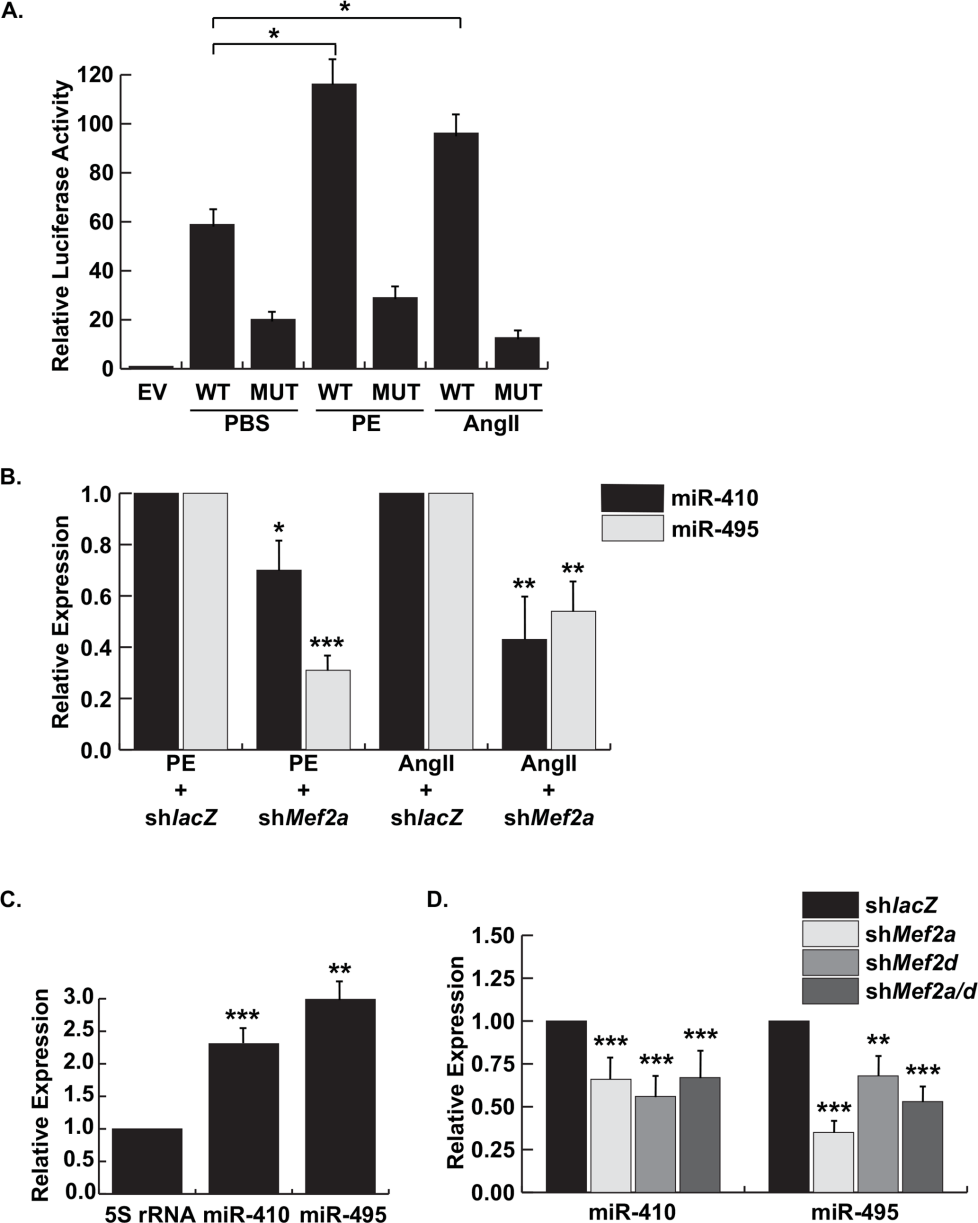
MEF2-dependent regulation of the *Gtl2-Dio3* proximal promoter and miRNAs in cardiac homeostasis and stress. *A*, Luciferase analysis of the wild type (WT) and MEF2 site mutant (MUT) *Gtl2-Dio3* proximal promoter in neonatal cardiomyocytes stimulated with PE or Ang II compared to control (PBS). *B*, Quantitative RT-PCR expression of miR-410 and miR-495 in control (sh*lacZ*) and MEF2A depleted (sh*Mef2a*) NRVMs treated with PE or Ang II, respectively. *C*, Quantitative RT-PCR expression of miR-410 and miR-495 in adult MEF2A knockout hearts. *D*, Quantitative RT-PCR expression of miR-410 and miR-495 in NRVMs depleted of MEF2A (sh*Mef2a*), MEF2D (sh*Mef2d*), or both (sh*Mef2a/d*) compared to control (sh*lacZ*). Error bars represent mean ± S.E. *, *p*<0.05; **, *p*<0.01; ***, *p*<0.001.

Having demonstrated the requirement of *Gtl2-Dio3* miRNAs in cardiomyocyte hypertrophy we next wanted to determine the mechanism by which this locus is regulated in stressed cardiomyocytes. Previously, we showed that the *Gtl2-Dio3* proximal promoter is active in unstressed NRVMs [13]. Therefore, we first examined the response of the *Gtl2-Dio3* proximal promoter to the above hypertrophic stimuli. As shown in Fig 4A, activity of the proximal promoter in PE- and Ang II-treated NRVMs was significantly increased in the presence of these pro-hypertrophic stimuli. Next, we asked whether MEF2 is required for this activation since it directly regulates the *Gtl2-Dio3* promoter and is an essential mediator of cardiac stress signaling. Therefore, we examined the activity of the *Gtl2-Dio3* proximal promoter harboring a mutation in the functional MEF2 site. Mutation of the MEF2 site significantly attenuated the response of the *Gtl2-Dio3* promoter to both stimuli, demonstrating that MEF2 is required for the upregulation of this locus in cardiac stress (Fig 4A). Conversely, depletion of MEF2A, the isoform that regulates the *Gtl2-Dio3* noncoding RNA locus in cardiomyocyte homeostasis, in NRVMs treated with either PE or Ang II significantly blunted the induction of endogenous miR-410 and miR-495 (Fig 4B).

To extend the above observations *in vivo*, we examined expression of miR-410 and miR-495 in adult MEF2A knockout hearts. Unlike perinatal MEF2A-deficient hearts, adult mutant mice do not display cardiomyocyte myofibrillar defects but instead develop a form of adult onset cardiomyopathy characterized by mitochondrial deficiency and conduction defects [17]. Paradoxically, we previously showed that an integrated MEF2-dependent reporter is highly upregulated in these adult mutant hearts indicating enhanced MEF2 activity in the absence of MEF2A. This activity presumably stems from post-transcriptional stimulation of MEF2D, a MEF2 isoform known to be involved in pathological cardiac remodeling [20]. Consistent with the cardiomyopathy phenotype and enhanced MEF2 activity in adult MEF2A-deficient hearts, miR-410 and miR-495 were found to be significantly upregulated (Fig 4C). Based on this result we asked whether MEF2D also regulates miR-410 and miR-495 expression in cardiomyocytes. As shown in Fig 4D, these *Gtl2-Dio3* miRNAs were sensitive not only to MEF2A but also to MEF2D depletion in unstressed NRVMs. Unexpectedly, combinatorial knockdown of MEF2A and D did not further reduce expression of these miRNAs suggesting that redundant regulation of this locus involves a more complicated mechanism than simple direct target effects.

## Discussion

In this report we demonstrate that the *Gtl2-Dio3* noncoding RNA locus is dynamically regulated in cardiomyopathies with diverse etiologies and function as pro-hypertrophic molecules in cardiomyocyte stress signaling *in vitro*. Expression analysis of the *Gtl2-Dio3* miRNAs, miR-410 and miR-495, in hypoxic, hypertrophic, and dystrophic cardiac environments, revealed robust induction of these miRNAs. In particular, myocardial infarction (hypoxia) in mice revealed marked differences in their spatio-temporal regulation in response to this cardiac injury. Furthermore, hypertrophic agonists potently activated the *Gtl2-Dio3* proximal promoter and induced expression of these miRNAs in isolated neonatal cardiomyocytes. Consistent with their upregulation, silencing of the miR-410 and miR-495 blunted cardiomyocyte hypertrophy *in vitro*. These data demonstrate regulation of the *Gtl2-Dio3* locus in cardiac diseases and that the noncoding RNAs expressed from this locus may be targeted to treat a variety of cardiomyopathies.

While other studies have reported the dysregulation of some *Gtl2-Dio3* miRNAs in cardiac disease models including myocardial infarction [21–24], these studies did not analyze expression of *Gtl2-Dio3* miRNAs in models of chronic cardiotoxic insult (Ang II), dystrophic cardiomyopathies (*mdx*, *DyW*), or in the temporal progression of myocardial infarction (MI). Moreover, comparative expression of these miRNAs in discrete regions of the heart, e.g. the remote myocardium and infarct area of the MI heart, whose pathological microenvironments are radically different, was not performed. Determining miRNA expression patterns in dystrophic cardiomyopathies is also a worthwhile endeavor as patients with DMD often die from cardiac complications [25–27]. Although cardiac failure in MDC1A congenital muscular dystrophy is less common, moderate cardiac abnormalities have been reported [28,29].

Our detailed *in vivo* expression analysis revealed two major findings. First, miR-410 and miR-495, and by extension the *Gtl2-Dio3* noncoding RNA cluster, were upregulated in all cardiac disease models examined. Given the diverse etiologies of these cardiomyopathies it is surprising that these miRNAs responded similarly to the seemingly disparate pathologies suggesting that the *Gtl2-Dio3* noncoding RNAs regulate a common remodeling process or pathway in heart disease. Second, different spatio-temporal expression patterns of both miR-410 and miR-495 were observed throughout the progression of MI. These expression differences may reflect the pathophysiology unique to the remote and infarcted areas [30]. Indeed, genome-wide transcriptome analyses have revealed region-specific (RM vs. IA) and chamber-specific differences in gene expression in infarcted hearts [31,32]. Through their post-transcriptional activity, variations in *Gtl2-Dio3* miRNA levels may help establish unique gene expression patterns that drive pathological remodeling events in distinct regions of the infarcted heart. Furthermore, the region-specific differences in *Gtl2-Dio3* miRNA profiles may reflect their expression associated with cell type composition in the remote and infarcted areas. Whereas the RM harbors predominantly myocytes undergoing extensive remodeling, the IA is scar tissue largely devoid of myocytes but consists of proliferating fibroblasts, myofibroblasts, and inflammatory cells. Given the role of miR-410 and miR-495 in cardiomyocyte proliferation it is tempting to speculate that their massive upregulation in the infarcted zone reflects expression in proliferating fibroblasts or myofibroblasts but their activation in this cell type remains to be determined.

Although overexpression of miR-410 or miR-495 is sufficient to promote robust proliferation of post-mitotic neonatal cardiomyocytes *in vitro*, their upregulation in Ang II-treated mice, in the remote zone of infarcted hearts, and in α-adrenergic stimulation of NRVMs did not stimulate myocyte proliferation. It is firmly established that cardiomyocytes are largely unable to proliferate when subjected to pathological stress signals but can be provoked to reenter the cell cycle only under specific conditions. Therefore, regardless of their overexpression in the heart, counter-regulatory mechanisms in cardiomyocytes subjected to chronic insults likely prevent these noncoding RNAs from inducing proliferation in cardiomyocytes. Alternatively, their levels, though elevated, may be insufficient to induce cardiomyocyte proliferation *in vivo*.

There is growing appreciation for the complex regulation of this maternally expressed, imprinted locus. Prior studies by us and others have demonstrated coordinate expression of the noncoding RNAs throughout the locus in ES cells, mouse embryos, and in cardiac and skeletal muscle [10,12,33]. In addition, our studies show that coordinate regulation is dependent on activation of the *Gtl2-Dio3* proximal promoter by MEF2 [12,13]. Contrary to these findings, some reports have described enhancers neighboring discrete miRNA clusters within the 200kb *Gtl2-Dio3* mega locus [34]. One of these enhancers is located upstream of the cluster containing both miR-410 and miR-495 [35]. Adding to the complexity of *Gtl2-Dio3* regulation are reports indicating either no significant change in expression or downregulation of some of its miRNAs, such as miR-495, in ischemic and dilated cardiomyopathies [23]. Apart from possible technical differences in the analysis methods or severity of disease, our data suggest that the *Gtl2-Dio3* noncoding RNA locus can be subject to differential regulation by the type of insult and in distinct regions of the injured heart either through transcriptional or post-transcriptional processes.

The MEF2-dependent regulation of the *Gtl2-Dio3* promoter in hypertrophic NRVMs is consistent with the ability of Ang II and the adrenergic hormone PE to stimulate MEF2 activity [36,37]. Interestingly, a recent study reported inhibition of MEF2 activity and cell death in myocytes exposed to adrenergic stimulation [38]. In this instance, however, myocytes were treated with isoproterenol, which is an adrenergic agonist that preferentially activates β_1_-type G protein-coupled receptors (GPCRs), whereas PE stimulates α_1_-type GPCRs. In the future it would be interesting to analyze the molecular mechanisms that regulate the *Gtl2-Dio3* promoter in cardiomyocytes downstream of various catecholaminergic signals.

In conclusion, we demonstrate that the *Gtl2-Dio3* miRNAs, miR-410 and miR-495, are upregulated in multiple models of cardiac disease, and that both of these miRNAs are regulated in a spatio-temporal specific fashion in myocardial injury. We also show that miR-410 and miR-495 function as prohypertrophic molecules such that their inhibition in stressed cardiomyocytes attenuates the pathological growth response. In the future, it will be of interest to examine regulation of the *Gtl2-Dio3* locus in additional cardiac diseases and other cardiac stress-inducing insults to determine whether or not the expression profile of its noncoding RNAs differs in each pathological condition. Additionally, identifying targets of miR-410, miR-495, and other miRNAs encoded by this locus in cardiomyopathies may lead to a better understanding of the pathways regulated by these molecules in pathological cardiac remodeling. Defining the roles of the miRNAs from this dynamically regulated noncoding RNA mega-locus in cardiomyopathies poses to be a challenging but potentially fruitful endeavor that may facilitate the development of strategies to target this locus in a disease-specific manner.
